# First attack of acute pancreatitis in Sweden 1988 – 2003: incidence, aetiological classification, procedures and mortality – a register study

**DOI:** 10.1186/1471-230X-9-18

**Published:** 2009-03-05

**Authors:** Birger Sandzén, Mats Rosenmüller, Markku M Haapamäki, Erik Nilsson, Hans C Stenlund, Mikael Öman

**Affiliations:** 1 Department of Surgical and Perioperative Sciences, Surgery, Umeå University Hospital, SE-901 85 Umeå, Sweden; 2 Department of Public Health and Clinical Medicine, Epidemiology and Public Health Sciences, Umeå University, SE-901 87, Umeå Sweden

## Abstract

**Background:**

Population-based studies suggest that the incidence of first attack of acute pancreatitis (FAAP) is increasing and that old age is associated with increased mortality. Beacuse nationwide data are limited and information on standardized mortality ratio (SMR) versus age is lacking, we wanted to describe incidence and mortality of first attack acute pancreatitis (FAAP) in Sweden.

**Methods:**

Hospital discharge data concerning diagnoses and surgical procedures and death certificate data were linked for patients with FAAP in Sweden. Mortality was calculated as case fatality rate (CFR), i.e. deaths per 1000 patients and SMR using age-, gender- and calendar year-specific expected survival estimates, and is given as mean with 95% confidence intervals. Data are presented as median values with 25% and 75% percentiles, means and standard deviations, or proportions. Proportions have been compared using the chi square test, Poisson-regression test or Fisher exact test. Location of two groups of ratio scale variables were compared using independent samples t-test or Mann-Whitney U-test.

**Results:**

From 1988 through 2003, 43415 patients (23801 men and 19614 women) were admitted for FAAP. Age adjusted incidence rose from 27.0 to 32.0 per 100000 individuals and year. Incidence increased with age for both men and women. At index stay 19.7% of men and 35.4% of women had biliary diagnoses, and 7.1% of men and 2.1% of women alcohol-related diagnoses. Of 10072 patients who underwent cholecystectomy, 7521 (74.7%) did so after index stay within the audit period. With increasing age CFR increased and SMR decreased. For the whole period studied SMR was 11.75 (11.34–12.17) within 90 days of index admission and 2.03 (1.93–2.13) from 91 to 365 days. Alcohol-related diagnoses and young age was associated with increased SMR. Length of stay and SMR decreased significantly during the audit period.

**Conclusion:**

Incidence of FAAP increased slightly from 1988 to 2003. Incidence increased and SMR declined with increasing patient age. Although the prognosis for patients with FAAP has improved it remains an important health problem. Aetiological classification at index stay and timing of cholecystectomy should be improved.

## Background

The diagnosis of acute pancreatitis is based on clinical features (abdominal pain) and elevation of plasma concentrations of pancreatic enzymes, if indicated supplemented by imaging techniques [1-4]. Gallstone disease is reported to account for approximately half of all cases with acute pancreatitis and alcohol abuse for 20–25%[5] with great variations between regions and over time.

Population-based studies suggest an increasing trend for incidence of first attack acute pancreatitis (FAAP)[5,6] although it is unclear to what extent this might reflect changes in population age. The presumption that old age is a risk factor for mortality[5,7] is based upon case fatality rate (CFR), which does not take into account mortality in the background population.

We have studied incidence, aetiological classification, procedures, and mortality for patients with FAAP in Sweden 1988–2003, using nationwide hospital discharge and death certificate data. Influence of age upon incidence and mortality was considered of particular importance.

## Methods

In Sweden patients are identified through a national registration number unique for each resident in Sweden. The Swedish National Board of Health and Welfare's Epidemiology Centre compiles data on individual hospital discharges in the Hospital Discharge Register (Patient administrativa registret, PAR)[8]. Since 1987, the register has included all Swedish hospitals. The record of each hospital stay contains diagnoses at discharge coded according to the Swedish version of the International Classification of Diseases (ICD), from 1987 through 1996 the 9th revision and from 1997 the 10th revision. Hospital stays are classified as emergent or elective. Surgical procedures are classified according to the Swedish version of Classification of Operations 1985 (revised 1988) and Classification of Surgical Procedures 1997. Underlying causes of death were obtained from Death Certificates, which have to be written for each patient after death in Sweden, are coded by the ICD-9 and ICD-10 classifications.

For all records reported to PAR a data control is run[8]. A quality control is made, that codes for different variables and dates have valid values and that compulsory variables (personal identification number, hospital and main diagnosis) are reported. Obviously incorrect data are corrected. In 2003, 0.9% of all diagnoses and 0.5% of acute somatic diagnoses was missing in hospital stays reported.

### Data acquisition

For the present study, data from PAR and from death certificate data were retrieved for all patients with a hospital admission from 1988 through 2003 for acute pancreatitis (577A in ICD-9 or K85 in ICD-10). Information on hospital admissions and procedures one year prior to and one year after index admission were collected for all patients.

### Definitions

First attack acute pancreatitis (FAAP) is defined as acute pancreatitis, without hospital visit with this diagnosis, during a minimum of one year preceding index admission. The aetiological diagnosis of FAAP refers to primary or secondary diagnoses during index admission; classified as biliary disease, alcohol-related disease or other (none of these) diagnoses. ICD codes in addition to K85.0 or 577A used for aetiological classification and for causes of death are given below:

#### Biliary disease

ICD 10: K80, K81, K82, K83

ICD 9: 574, 575, 576

#### Gallstone disease

ICD 10: K80

ICD 9: 574

#### Alcohol-related disease

ICD 10: K70.0, K70.2-4, K70.9, F10.0-9, F9.0-9, G31.2, T51.0, Z72.1

ICD 9: 571A-D, 571F, 572W, 291A-F, 291W, 291X, 304H, 305A. 305X, 303, 9800

Procedures were classified as biliary procedures or operations on pancreas (codes introduced in 1997 with capital letters).

#### Biliary procedures

Open cholecystectomy JKA20, 5350, -51, -52, -56, -57, -59

Laparoscopic cholecystectomy JKA21, 5353

Intraoperative cholangiography TJK00, TJK01, 0057

Intraoperative cholangioscopy JKB20, TJK21, 0057

Common bile duct exploration JKB00, JKB01, 5300, -02, -04, -06, -09, 5351, -52, -56, -57

#### Operations on pancreas

Exploration, biopsy of pancreas JLA, 5580, -81

Incision, drainage of pancreas JLB, 5500, -02, 0061, -66

Resection of pancreas JLC, 5510, -11, -12, -14, -15, -16

Other operations on pancreas, open or laparoscopic JLW, 5520, -21, 5590, -91, -92, -93, 5599

### Statistical analysis

Data are presented as median values with 25% and 75% percentiles, means and standard deviations, or proportions. Proportions have been compared using the chi square test, Poisson-regression test or Fisher exact test when appropriate. Location of two groups of ratio scale variables were compared using independent samples t-test or Mann-Whitney U-test. Mortality was calculated as case fatality rate (CFR), i.e. deaths per 1000 patients and standardized mortality ratio (SMR) using age-, gender- and calendar year-specific expected survival estimates from Statistics Sweden[9]. SMR is given as mean with 95% confidence intervals. Calculations were performed with SPSS 15.0 (SPSS Inc. Chicago, IL, USA). A p-value less than 0.05 was considered significant.

### Ethics

Ethical approval for the study was obtained from the Regional Research Ethics Committee of the University of Umeå, Sweden (Dnr 06-027M) before commencement of the project.

## Results

### Incidence

From 1 January 1988 through 31 December 2003 totally 43415 patients, 23801 men and 19614 women were admitted for FAAP in Sweden. Table 1 shows number of patients per age group. There is a highly significant (p < 0.001) time-dependent difference in age distribution. This is more evident in Table 2 illustrating age specific incidences, in which the crude overall incidence and incidence adjusted to the Swedish population of 1988–1992 are given. Within each time period, the incidence of first attack acute pancreatitis increased with patients' age. The age adjusted incidence increased significantly for women (p < 0.001) and for men and women together (p < 0.001) but not for men (p > 0.05). For both men and women above the age of 70 years, the adjusted age specific incidence increased significantly (p < 0.001) with time. Considering all patients, the adjusted incidence rose modestly, from 27.0 per 100000 individuals per year 1988–1992 to 32.0 1998–2003. This increase was entirely due to a significant (p < 0.001,) rise in incidence for women, from 21.1 to 31.0 per 100000 per year.

**Table 1:** Number of patients per age group with first attack of acute pancreatitis versus time period

Age (years)	Time period 1988–1992	Time period 1993–1997	Time period 1998–2003	**Time period 1988–2003**
0 – 49	4341 (37.5)	4372 (31.0)	5109 (28.9)	13822 (31.8)
50 – 59	1792 (15.5)	2448 (17.3)	3259 (18.4)	7499 (17.3)
60 – 69	2046 (17.7)	2435 (17.2)	2968 (16.8)	7449 (17.2)
70 – 79	2055 (17.7)	2800 (19.8)	3484 (19.7)	8339 (19.2)
80 +	1356 (11.7)	2071 (14.7)	2879 (16.3)	6306 (14.5)

**All**	**11590 (100)**	**14126 (100)**	**17699 (100)**	**43415 (100)**

Percent of all patients in each time period within brackets

**Table 2:** Age specific incidence per 100 000 individuals of first attack acute pancreatitis at index stay 1988–2003.

	Incidence women			Incidence men			Total incidence		
**Age (years)**	**1988–1992**	**1993–1997**	**1998–2003**	**1988–1992**	**1993–1997**	**1998–2003**	**1988–1992**	**1993–1997**	**1998–2003**
			
0 – 49	10.0	13.0	14.6	20.0	17.0	15.3	15.1	15.0	15.0
50 – 59	27.4	38.3	40.1	53.3	53.9	48.3	40.3	46.2	44.2
60 – 69	35.0	48.2	52.2	60.6	73.9	66.1	47.2	60.5	58.9
70 – 79	47.9	66.8	73.3	70.3	90.1	96.3	57.7	77.0	83.5
80 +	67.1	89.5	94.3	85.6	119.9	125.7	73.6	100.1	105.4
			
**Crude**	21.1	28.8	31.9	33.1	35.3	34.4	27.0	32.0	33.1
**Adjusted**	21.1	28.5	31.0	33.1	34.9	33.0	27.0	31.7	32.0

Crude = incidence considering all patients and population at time period. Adjusted = incidence adjusted to the Swedish population at 1988–1992.

### Aetiological diagnoses

In Table 3 the aetiological diagnoses and age at index stay for men and women with FAAP is shown. Considering all patients, men were younger than women, 58 (45–72) versus 63 (46–77) years, median and (25–75 percentiles). Biliary diagnoses were identified for 19.7% of all men and for 35.4% of women and alcohol-related diagnoses for 7.1% and 2.1%, respectively, including patients with both alcohol related and biliary diagnoses (N = 51). Patients with gallstone disease (N = 10348) constitute 88.5% of all patients with biliary diagnoses. As evident from Table 3, patients with alcohol-related acute pancreatitis were significantly younger than patients with biliary diagnoses. Most strikingly, 68.4% of all patients had no specified aetiological diagnosis at index stay.

**Table 3:** Aetiological diagnosis and age of patients with first attack acute pancreatitis at index stay 1988 – 2003

	Number at index stay			Median age		
**Diagnosis**	**Men**	**Women**	**Total**	**Men**	**Women**	**Total**
		
Biliary *	4693 (19.7)	6943 (35.4)	11636 (26.8)	68 (54–77)	63 (46–76)	65 (50–77)
Alcohol-related **	1691 (7.1)	410 (2.1)	2101 (4.8)	49 (42–57)	49 (43–58)	49 (42–58)
Other	17417 (73.2)	12261 (62.5)	29678 (68.4)	56 (44–70)	64 (47–77)	59 (45–73)
		
**Total**	**23801 (100)**	**19614 (100)**	**43415 (100)**	**58 (45–72)**	**63 (46–77)**	**60 (45–74)**

In number at index, percent and in median age, 25–75 percentiles within brackets. * Patients with gallstone disease (N = 10348) constitute 88.5% of all patients with biliary diagnoses. ** Including patients with alcohol related and biliary diagnoses (N = 51)

### Recurrent attack of acute pancreatitis after FAAP

Of all 43415 patients with FAAP 7329 (16.9%) had at least one additional attack of (recurrent) acute pancreatitis within one year of index admission and of these 1246 (2.9%) had two episodes. Recurrent attacks were less common after biliary FAAP, 1103 of 11636 (9.4%), than after alcohol-related, 402 of 2101 (19.1%) and "other" FAAP, 5824 of 29678 (19.6%), p < 0.001 for both comparisons.

### Length of hospital stay

Table 4 shows length of hospital stay for all patients with FAAP during the time period covered by this audit. A significant (p < 0.001, Mann-Whitney test) decrease in length of stay with time is noted both for men, for women, and for both gender together. There was no significant difference (p = 0.46) between length of stay for men and women.

**Table 4:** Length of hospital stay of patients with first attack acute pancreatitis at index stay 1988–2003 in different time periods.

Time period	Men *	Women *	Total *
1988–1992	6 (3 – 10)	7 (4 – 11)	6 (4 – 11)
1993–1997	6 (3 – 10)	6 (4 – 10)	6 (3 – 10)
1998–2003	5 (3 – 9)	5 (3 – 9)	5 (3 – 9)

**Total period**	**6 (3 – 10)**	**6 (4 – 10)**	**6 (3 – 10)**

Length of stay in days, median (p25 – p75). * Significant (p < 0.001) decrease in length of stay in different time periods in men, women and total. No difference between men and women.

### Procedures

Table 5 indicates for each group cholecystectomies performed during three periods: one year preceding index stay, at index stay, and one year after index stay. 10072 of all 43415 patients with FAAP (23.2%) underwent cholecystectomy from one year before index admission until one year after index admission. However, only 1932 or 4.5% of all patients had a cholecystectomy during index stay. Of 11636 patients with biliary diagnoses 6386 (55.7%) had a cholecystectomy, which for 1648 patients (14.2%), was performed during the index stay. Cholecystectomy rate rose modestly with time, from 18.8% to 26.9% of all patients, mainly because of an increased cholecystectomy rate after the index stay.

**Table 5:** Patients with FAAP who underwent cholecystectomy in relation to index stay

Diagnoses (N)	Before (%)	During (%)	After (%)	Total (%)
Biliary diagnoses (11636)	219 (1.9)	1648 (14.2)	4619 (39.7)	6486 (55.7)
Alcohol-related diagnoses (2101)	6 (0.3)	10 (0.5)	21 (1.0)	37 (1.8)
Other diagnoses (29678)	394 (1.3)	274 (0.9)	2881 (9.7)	3549 (11.9)
All diagnoses (43415)	619 (1.4)	1932 (4.5)	7521 (17.3)	10072 (23.2)

**Time periods (N)**				

1988–1992 (11590)	121 (1.0)	423 (3.6)	1639 (14.1)	2183 (18.8)
1993–1997 (14126)	224 (1.6)	597 (4.2)	2316 (16.4)	3137 (22.2)
1998–2003 (17699)	274 (1.5)	912 (5.2)	3567 (20.2)	4753 (26.9)

N = all patients admitted with FAAP. Before = Within one year before index stay, During = During index stay, After = Within one year after index admission.

During the audit period 3506 patients (8.1%) underwent sphincterotomy (Table 6). The overall rise in sphincterotomy rate (from 2.8 to 12.1% of all patients) was mainly due to an increase from 1.2% 1988–92 to 7.7% 1999–2003 during index stay. A slight decrease in exploration of the common bile duct was seen, from 5.0% of all patients 1988–1992 to 2.9% 1998–2003 (Table 7). Pancreatic surgery (drainage, exploration and resection) was done on 1346 of all 43415 patients (3.1%) with no time trend (Table 8).

**Table 6:** Patients with FAAP who underwent endoscopic sfincterotomy in relation to index stay

Diagnoses (N)	Before (%)	During (%)	After (%)	Total (%)
Biliary diagnoses (11636)	95 (0.8)	1538 (13.2)	572 (4.9)	2205 (18.9)
Alcohol-related diagnoses (2101)	1 (0.0)	7 (0.3)	7 (0.3)	15 (0.7)
Other diagnoses (29678)	101 (0.3)	524 (1.8)	659 (2.2)	1284 (4.3)
All diagnoses (43415)	197 (0.5)	2069 (4.8)	1238 (2.9)	3504 (8.1)

**Time periods (N)**				

1988–1992 (11590)	15 (0.1)	136 (1.2)	172 (1.5)	323 (2.8)
1993–1997 (14126)	69 (0.5)	568 (4.0)	398 (2.8)	1035 (7.3)
1998–2003 (17699)	113 (0.6)	1365 (7.7)	668 (3.8)	2146 (12.1)

N = all patients admitted with FAAP. Before = Within one year before index stay, During = During index stay, After = Within one year after index admission.

**Table 7:** Patients with FAAP who underwent common bile duct exploration in relation to index stay

Diagnoses (N)	Before (%)	During (%)	After (%)	Total (%)
Biliary diagnoses (11636)	50 (0.4)	454 (3.9)	501 (4.3)	1005 (8.6)
Alcohol-related diagnoses (2101)	0 (0.0)	0 (0.0)	3 (0.1)	3 (0.1)
Other diagnoses (29678)	67 (0.2)	79 (0.3)	362 (1.2)	508 (1.7)
All diagnoses (43415)	117 (0.3)	533 (1.2)	866 (2.0)	1516 (3.5)

**Time periods (N)**				

1988–1992 (11590)	45 (0.4)	191 (1.6)	347 (3.0)	583 (5.0)
1993–1997 (14126)	36 (0.3)	157 (1.1)	225 (1.7)	428 (3.0)
1998–2003 (17699)	36 (0.2)	185 (1.0)	284 (1.6)	505 (2.9)

N = all patients admitted with FAAP. Before = Within one year before index stay, During = During index stay, After = Within one year after index admission.

**Table 8:** Patients with FAAP who underwent operations on pancreas in relation to index stay

Diagnoses (N)	Before (%)	During (%)	After (%)	Total (%)
Biliary diagnoses (11636)	6 (0.1)	111 (1.0)	127 (1.1)	244 (2.1)
Alcohol-related diagnoses (2101)	1 (0.0)	13 (0.6)	23 (1.1)	37 (1.8)
Other diagnoses (29678)	84 (0.3)	400 (1.3)	581 (2.0)	1065 (3.6)
All diagnoses (43415)	91 (0.2)	524 (1.2)	731 (1.7)	1346 (3.1)

**Time periods (N)**				

1988–1992 (11590)	31 (0.3)	143 (1.2)	207 (1.8)	381 (3.3)
1993–1997 (14126)	27 (0.2)	188 (1.3)	226 (1.6)	441 (3.1)
1998–2003 (17699)	33 (0.2)	193 (1.1)	298 (1.7)	524 (3.0)

N = all patients admitted with FAAP. Before = Within one year before index stay, During = During index stay, After = Within one year after index admission.

### Mortality and cause of death

**Figure 1:**
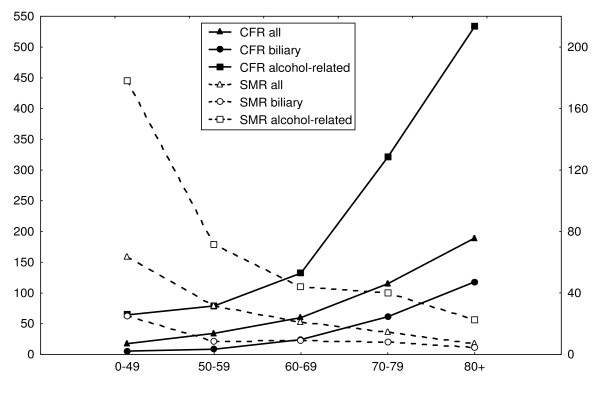
**Mortality in acute pancreatitis within 90 days CFR/1000 and SMR**. Case fatality rate (CFR) on the left Y-axis and standardized morality ratio (SMR) on the right Y-axis. Age in years on X-axis.

The CFR and SMR for patients in the three aetiological groups declined significantly for each period during the audit, see Table 9. As shown in Table 10, 3101 patients (7.1%) died within 90 days of admission for FAAP and a further 1604 (3.7%) 91 to 365 days thereafter, yielding SMR:s of 11.75 (11.34–12.17) and 2.03 (1.93–2.13), respectively. The SMR for patients with an alcohol-related FAAP was 58.70 (50.96–67.29) 0–90 days after index admission, compared to 5.84 (5.34–6.37) for patients in the biliary group, and 8.77 (8.37–9.19) for patients without aetiological diagnosis (Table 11, 12 and 13). For all patients together and for the three groups separately, SMR declined with patients' age. This contrasts to case fatality rates (CFR), which increased with patients' age, outlined in Figure 1.

**Table 9:** CFR and SMR for all groups of diagnoses in different time periods

Patients	Deaths within 90 days			Deaths 91–365 days	
**Time periods**	**CFR**	**SMR**	**Deaths**	**SMR**	**Deaths**
		
1988–1992	82.4	16.82 (15.65–18.06)	757	2.32 (2.07–2.59)	318
1993–1997	75.8	12.52 (11.84–13.23)	1252	2.19 (2.02–2.36)	658
1998–2003	61.7	9.25 (8.71–9.82)	1092	1.77 (1.64–1.92)	628

SMR (standardized mortality ratio) given as median (confidence intervals 95%), CFR (case fatality rate) is given as deaths per 1000 patients.

**Table 10:** CFR and SMR for all groups of diagnoses in different age groups

Patients		Deaths within 90 days			Deaths 91–365 days	
**Age**	**Patients**	**CFR**	**SMR**	**Deaths**	**SMR**	**Deaths**
		
0–49	13822	17.6	63.51 (55.77–72.01)	243	15.51(13.31–17.96)	178
50–59	7499	34.3	31.67 (27.91–35.79)	257	7.48(6.43–8.64)	182
60–69	7449	60.1	20.92 (19.02–22.95)	448	4.30 (3.80–4.83)	276
70–79	8339	115.0	14.60 (13.69–15.55)	959	2.24 (2.03–2.46	441
80 +	6306	189.3	7.24 (6.84–7.66)	1194	1.07 (0.98–1.16)	527
		
**Total**	**43415**	**71.4**	**11.75 (11.34–12.17)**	**3101**	**2.03 (1.93–2.13)**	**1604**

SMR (standardized mortality ratio) given as median (confidence intervals 95%), CFR (case fatality rate) is given as deaths per 1000 patients.

**Table 11:** CFR and SMR for biliary diagnoses in different age groups

Patients		Deaths within 90 days			Deaths 91–365 days	
**Age**	**Patients**	**CFR**	**SMR**	**Deaths**	**SMR**	**Deaths**
		
0–49	2910	5.5	24.78 (14.15–40.24)	16	9.29 (5.50–14.68)	18
50–59	1775	8.5	8.32 (4.65–13.73)	15	3.88 (2.40–5.94)	21
60–69	2095	24.3	8.83 (6.58–11.61)	51	2.60 (1.89–3.48)	45
70–79	2722	61.4	7.95 (6.79–9.26)	167	1.60 (1.31–1.95)	101
80 +	2185	118.1	4.48 (3.95–5.06)	258	0.97 (0.83–1.12)	167
		
**Total**	**11687**	**43.4**	**5.84 (5.34–6.37)**	**507**	**1.35 (1.21–1.50)**	**352**

SMR (standardized mortality ratio) given as median (confidence intervals 95%), CFR (case fatality rate) is given as deaths per 1000 patients.

**Table 12:** CFR and SMR for alcohol-related diagnoses in different age groups

Patients		Deaths within 90 days			Deaths 91–365 days	
**Age**	**Patients**	**CFR**	**SMR**	**Deaths**	**SMR**	**Deaths**
		
0–49	1069	64.5	177.88 (138.39–225.12)	69	19.76 (12.52–29.66)	23
50–59	594	79.1	71.42 (52.47–94.98)	47	11.14 (6.98–16.87)	22
60–69	302	132.5	44.13 (31.53–60.10)	40	4.41 (2.28–7.71)	12
70–79	106	320.8	40.02 (27.71–5.92)	34	1.18 (0.24–3.44)	3
80 +	30	533.3	22.62 (12.92–36.74)	16	0.00 (0.00–0.00)	0
		
**Total**	**2101**	**98.0**	**58.70 (50.96–67.29)**	**206**	**5.70 (4.35–7.34)**	**60**

SMR (standardized mortality ratio) given as median (confidence intervals 95%), CFR (case fatality rate) is given as deaths per 1000 patients.

**Table 13:** CFR and SMR for other diagnoses in different age groups

Patients		Deaths within 90 days			Deaths 91–365 days	
**Age**	**Patients**	**CFR**	**SMR**	**Deaths**	**SMR**	**Deaths**
		
0–49	9854	16.1	56.86 (48.36–66.41)	159	16.45 (13.82–19.43)	138
50–59	5142	38.1	34.58 (29.91–39.77)	196	8.17 (6.87–9.65)	139
60–69	5064	70.5	24.15 (21.71–26.79)	357	4.94 (4.31–5.64)	219
70–79	5520	137.9	17.33 (16.12–18.61)	761	2.56 (2.29–2.85)	337
80 +	4098	225.7	8.67 (8.12–9.24)	925	1.12 (1.01–1.25)	360
		
**Total**	**29678**	**80.8**	**13.79 (13.24–14.35)**	**2398**	**2.29 (2.16–2.42)**	**1193**

SMR (standardized mortality ratio) given as median (confidence intervals 95%), CFR (case fatality rate) is given as deaths per 1000 patients.

Cause of death for all patients who died within one year of index admission is given in Table 14. According to death certificates, biliary diagnoses were the underlying causes of death for 10.5% of all patients who died within 90 days of index admission, whereas alcohol-related diagnoses accounted for 1.1%, and all other diagnoses for 88.4%. From three to twelve months after index admission 3.7% of all patients who died had biliary or alcohol-related diagnoses as underlying causes of death.

**Table 14:** Cause of death within 90 days and 91–365 days from index admission

Underlying cause of death within 90 days				
**Diagnoses**	**1988–1992**	**1993–1997**	**1998–2003**	**1988–2003**

Biliary diagnoses	94 (9.8)	113 (10.7)	119 (10.9)	326 (10.5)
Alcohol related diagnoses	5 (0.5)	13 (1.2)	17 (1.6)	35 (1.1)
Other diagnoses	858 (89.7)	926 (88.0)	956 (87.5)	2740 (88.4)

**Total**	**957 (100)**	**1052 (100)**	**1092 (100)**	**3101 (100)**

**Underlying cause of death within 91–365 days**				

**Diagnoses**	**1988–1992**	**1993–1997**	**1998–2003**	**1988–2003**

Biliary diagnoses	14 (3.4)	15 (2.7)	11 (1.8)	40 (2.5)
Alcohol related diagnoses	2 (0.5)	9 (1.6)	8 (1.3)	19 (1.2)
Other diagnoses	400 (96.2)	536 (95.7)	609 (97.0)	1545 (96.3)

**Total**	**416 (100)**	**560 (100)**	**628 (100)**	**1604 (100)**

Number of patients with percent within brackets.

## Discussion

In this register study we found a crude incidence of first attack acute pancreatitis (FAAP) of 27–33 per 100000 individuals per year. Biliary aetiology was more common among women than men, whereas the reverse was true for alcohol-related diagnoses. Two thirds of patients with FAAP lacked an aetiological diagnosis at index stay. SMR was raised twelve fold within 90 days of index admission and decreased with patients' age in contrast to CFR, which increased with increasing age.

We have utilized nation-wide data from all patients with FAAP in Sweden 1988–2003. Personal identification numbers make it possible to trace patients over time and to link hospital discharge data and death certificate data. The clinical features of acute pancreatitis (abdominal pain) together with elevation of plasma concentrations of pancreatic enzymes are well known to surgeons and physicians taking care of emergency patients, and the diagnostic validity in our audit can be considered high.

As main measure of mortality we used SMR, thereby adjusting for the mortality of the background population. Like most register studies, we have limited clinical information of patients' clinical conditions. Aetiological diagnoses in our audit refer to classification at index discharge, as treatment of acute pancreatitis should be based upon knowledge of aetiology (and disease severity) at index stay.

The Swedish hospital discharge register do not take into account outpatient procedures. This is unlikely to introduce any substantial error in cholecystectomy rate as day-case cholecystectomy was of limited use during the period studied. Of all cholecystectomies done in 2005 12% were done as day-cases. This may be deduced by comparing in-hospital cholecystectomy and over-all cholecystectomy figures for Sweden[8,10]. Some sphincterotomies may also have been performed as ambulatory procedures, and therefore not registered before or after index stay, most likely in the latter part of the audit period.

In analyses of acute pancreatitis it is of importance to differentiate between first and recurrent attack[4,11,12]. We found that 16.9% of all patients with FAAP had a recurrent attack of acute pancreatitis within one year of FAAP. Recurrent pancreatitis was less common (9.4%) for patients with biliary pancreatitis, most likely attributable to the fact that 55.7% of these patients had a definitive treatment (cholecystectomy) within one year of index admission for FAAP.

Incidence of FAAP comparable to the one found in this audit has been described for other non-UK studies[5,13,14]including Sweden[11,12], whereas a lower incidence is reported from UK[5,15] and the Netherlands[16]. Population-based studies[5,6,14] indicate an increasing incidence with time, particularly for women. In the present 16-year audit, incidence increased slightly but significantly for women but not for men. During the last six years of our audit, crude incidence for men and women differed by less than 10%.

Studies reporting age adjusted incidence rates have found that incidence increases with age[13,14,17,18]. We observed a six-fold increase in age standardized incidence from patients 0–49 years of age to patients 80 years and above. For patients above the age of 70 years, the incidence increased significantly during the audit period. One might speculate that this could be due to a true increase of biliary disease in elderly or to a reduction in cholecystectomy rate. We have no data concerning change in gallstone prevalence during the period studied and no specific data for different age groups. There is a falling cholecystectomy rate during the 80s, followed by a noticeable increase after introduction of laparoscopic cholecystectomy in the early 90s[19,20]. However, according to Danish data this increase does not hold for older ages, where the cholecystectomy rate is decreasing[19].

As in previous studies, biliary aetiology was more common in women than in men, whereas the reverse was true for alcohol-related aetiology. The most striking finding in our audit was, however, that 68.4% of all patients with first attack acute pancreatitis lacked an aetiological diagnosis at index stay. This is at variance with the UK guidelines according to which the aetiology of acute pancreatitis should be determined in at least 80% of cases and no more than 20% should be classified as idiopathic[2]. As treatment should be governed by aetiology and severity of disease at index stay, substandard aetiological classification may lead to sub-optimal treatment.

Previous studies[3,21] have demonstrated that some 85% of all patients with FAAP might be expected to have moderate or mild pancreatitis. According to UK guidelines[2], definitive treatment at index stay is motivated in mild gallstone pancreatitis, and unless the patient is unfit for surgery this will be by cholecystectomy. However, expectant management with interval cholecystectomy has been considered appropriate for most patients with mild to moderate pancreatitis and an improving clinical course[3]. Patients with mild acute biliary pancreatitis who are poor candidates for surgery and patients with severe gallstone FAAP in combination with cholangitis, jaundice, or a dilated common bile may benefit from (early) ERCP at index stay[2,3]. In the present audit, approximately five per cent of all patients underwent cholecystectomy and an equal percentage ERCP at index stay, whereas the majority of all cholecystectomies were done after index stay. Obviously, a scrutiny of treatment for mild gallstone acute pancreatitis in Sweden is motivated. It has recently been shown that feed-back to surgeons may increase adherence to guidelines in this field[22].

Previous reports[5] describe that the mortality of patients with FAAP increases with age. Also in the present study, CFR increased with patients' age. However, SMR is a better measure of the excess mortality risk caused by the disease studied, as it adjusts for the mortality of background population. We found that SMR decreased with age irrespectively of aetiological diagnosis of FAAP. This seems to indicate that the vulnerability of the pancreas declines with age. In this connection, the finding of Sotoudehmanesh et al. [23] that the risk for post-ERCP pancreatitis is reduced in patients above the age of 60 years is of interest.

In the present study SMR was elevated twelve-fold 0–90 days after admission for first attack acute pancreatitis and doubled 91–365 days after admission, considering all patients together. For patients with biliary or alcohol-related diagnoses the SMR within 90 days was 5.84 and 58.70, respectively. It has been noted previously that patients with acute pancreatitis of alcohol-related aetiology are more likely than patients with gallstone pancreatitis to develop severe disease[24]. However, similar mortality rates have been reported for acute pancreatitis of different origin[5,25]. These conflicting results are best explained by the fact that the same CFR value is associated with a higher SMR value in younger patients (with alcohol-related acute pancreatitis) compared to older patients (with gallstone associated pancreatitis).

The over-all importance of aetiological factors behind severe FAAP in Sweden is best described by our finding that biliary diagnoses were 10 times more frequent than alcohol-related diagnoses as the underlying cause of death within 90 days.

The need to identify cost-effective strategies to prevent and treat acute pancreatitis has been emphasized [26-28] and our study underlines this. In agreement with previous studies [5] we found that CFR (and SMR) decreased in recent years. This observation and the shortening of the length of stay during the audit period seem to indicate that treatment of FAAP in Sweden has improved.

## Conclusion

An overall incidence of 33 per 100000 inhabitants per year and a mortality rate twelve times higher than for the background population during the first 90 days after hospital admission make FAAP a health care problem of great importance in Sweden. There is room for improvement of aetiological diagnosis at index stay and for management of acute pancreatitis of biliary origin.

## Abbreviations

SMR: Standardized mortality ratio; CFR: case fatality rate; AP: acute pancreatitis; FAAP: first attack acute pancreatitis; PAR: Hospital Discharge Registe; ICD-9, ICD-10: International Classification of Diseases 9^th ^and 10^th ^edition; LOS: length of hospital stay; ERCP: endoscopic retrograde cholangiopancreatiography.

## Competing interests

The authors declare that they have no competing interests.

## Authors' contributions

The study was planned by BS, MR, MMH, EN and MÖ. HS participated in the design of the study and performed the statistical analyses. BS, EN and MÖ drafted the manuscript. All authors have read and approved the final the manuscript.

## Pre-publication history

The pre-publication history for this paper can be accessed here:

http://www.biomedcentral.com/1471-230X/9/18/prepub
